# Sexual orientation, gender identity and gender expression-based violence in Catalan universities: qualitative findings from university students and staff

**DOI:** 10.1186/s13690-021-00532-4

**Published:** 2021-02-04

**Authors:** Elena María Gallardo-Nieto, Aitor Gómez, Regina Gairal-Casadó, María del Mar Ramis-Salas

**Affiliations:** 1 Universitat Rovira i Virgili, Tarragona, Spain; 2 Universitat de Barcelona, Barcelona, Spain

**Keywords:** Violence, Discrimination, Social problem, Health education, Universities, Sexual orientation, Gender identity

## Abstract

**Background:**

Hate crimes have raised in Spain and the gender and sexuality-based conflicts persist worldwide which leads to this problem having an effect on health and wellbeing. Following a focus of transforming Higher Education Institutions, this research analysed the problem that affects undergraduate students in six Spanish universities. The research goal is to improve the life quality of lesbian, gay, bisexual, transgender, queer and intersex university students, breaking the silence that exists around the violence that this group suffer in Catalonia, Spain.

**Methods:**

Following the Communicative Methodology, this study has identified violence based on sexual orientation, gender identity or gender expression in the target universities and provided guidelines to improve anti-discrimination protocols. A qualitative method has reached experiences of university students, heads of equality commissions, professors and administrative staff regarding this conflict. Focussing on the qualitative research tools, 30 semi-structured interviews were conducted with university students and staff around issues related to the violence against lesbian, gay, bisexual transgender, queer and intersex students: 1) perception of violence and discrimination, 2) institutional measures, 3) actions against violence. An analysis of exclusionary and transformative dimensions was used to identify emergent themes.

**Results:**

We have identified two dimensions for the analysis given their impact in contributing or overcoming violence: exclusionary and transformative. A wide range of forms of violence on the grounds of sexual orientation, gender identity and gender expression perpetrated at universities have been identified as exclusionary facts and described by participants in the study. Equality commissions have not received reports of violence based on sexual orientation, gender identity or gender expression, and university staff shows certain unfamiliarity regarding the measures and politics to prevent and intervene in cases of violence against the lesbian, gay, bisexual, transgender, queer and intersex community. Among the results identified as transformative are the ways through which actions of lesbian, gay, bisexual, transgender, queer and intersex groups against violence and the professors’ commitment to intervene have a relevant impact on student’s wellbeing. An improvement and implementation of anti-discrimination protocols with mandatory applicability has also been documented.

**Conclusions:**

Findings highlight the need of collecting more evidence that contributes to the improvement of protocols, measures and politics to protect all the members of the university community. A better understanding of violence based on sexual orientation, gender identity and gender expression in HEI’s may guide national and international governments to improve the health and well-being of lesbian, gay, bisexual, transgender, queer and intersex persons.

## Background

Violence based on sexual orientation, gender identity or gender expression is present in our society and within the university community [1–3]. Numerous international studies have shown that the lesbian, gay, transgender, queer and intersex (LGBTQI+) community have more risk and probabilities to suffer sexual discrimination or harassment during their university trajectory [2, 3]. Furthermore, the risk of being object of violence increases in the case of transgender students [4–6]. National politics and international agendas have given priority to the legislation and regulation to end with LGBTQI-phobia [7, 8], even though the risk of suffering sexual harassment is still higher in the case of sexual minorities [1, 6, 9, 10]. The case of Spanish universities reflects the international panorama in relation to the LGBTQI+ academic community [11]. Educating in diversity, tolerance and acceptance towards diversity is crucial, but international reports show that this is still a pending issue in Spain [12]. The lack of literature and research about how this phenomenon affects Higher Education Institutions (HEI’s) is striking and generates a significant silence towards the situation of the LGBTQI+ community and the consequences of LGBTQI-phobia in their personal, academic and health status. As a response to this reality, the research *Uni4freedom* seeks to contribute to breaking the silence that goes along the LGBTQI+ community at HEI’s, improving the quality of life of the academic community and the struggle against violence based on sexual orientation, gender identity and gender expression in the Catalan context.

The main challenge when studying the discrimination against the diversity of sexual orientations, gender identities and expressions is the diversity of violence manifestations. A change in the ways violence against the LGBT community is manifested, has been shown in the literature in the last years, shifting towards more subtle and unnoticed manifestations [6]. Verbal forms of violence [9, 13, 14], homophobic jokes [10, 15, 16], anti-LGBT paintings, graffities and threats [3, 13], social distance [10, 15, 17], not-inclusive or acceptance spaces [18] and possible risk of suffering from unprotected forms of sex and AIDS’ contagion [19] have been identified as advanced forms of violence based on sexual orientation or gender identity or expression. On the one hand, the case of a hostile environment is conditioning the free expression of the felt gender identity and sexual orientation [9, 18–22]. On the other hand, all these forms and manifestations of violence can generate a response of internalization and normalization of the homophobic actions, perpetuating the violence and affecting negatively in the life quality and wellbeing of the LGBT+ academic community [3, 16, 22–25].

According to the findings in the scientific literature, violence and discrimination based on sexual orientation and gender identity and expression, has consequences in three spheres of the LGBT university students’ life. Firstly, it affects the health status, both physically and mentally. It has been shown that LGBT students present higher symptoms of depression and anxiety [9, 13] and suffer various forms of physical ache [10, 16]. Secondly, it affects their academic performance, presenting a lower grade in average in comparison to hetero-cis students [26]. This difference of academic results has been analysed as an aftereffect of LGBT stigmatization creating difficulty to focus on their studies [19, 27], having further consequences in their future possibilities and academic success [24]. Thirdly, these realities of LGBT-phobia in HEI’s promote exclusionary climates and negatively affects the cohesion and relationality of sexual minority students [22]. The seen or suffered experiences of harassment or discrimination can generate a feeling of isolation and exclusion [6, 26] which can be reinforced by the institutional invisibility of LGBT perspectives and role models [13].

Evidence shows six different axes to prevent and intervene in front of violence based on sexual orientation and gender identity and expression within university institutions. Visibility of the LGBTQI+ collective and their situation in the university scope is key to prevent LGBTQI-phobia. Besides, generating profound awareness of university members is necessary to be able to face the problem of LGBT-phobia [1, 28]. In order to make this awareness effective and reach the whole academic community, the inclusion of LGBT literature in academic curriculums has the highest impact in the reduction of LGBT-phobia [18, 28]. Another protective factor for the prevention and intervention in cases of violence based in sexual orientation, gender identity and gender expression is the explicit institutional support towards the LGBTQI+ community through the implementation of politics and strategies towards the reduction of hetero-sexism [6, 9, 29, 30].

This article presents qualitative evidence about how to overcome this form of gender inequality in a very relevant social institution. We have chosen a qualitative method in order to delve into the complexities of suffering or witnessing violence at HEI’s and the possibilities of intervention that participants identify in their own interpretation. This methodological choice aims to reinforcing the knowledge and contrasting the depth and complexities of the qualitative findings of the project. Very important issues to be considered when preparing policies for the prevention and intervention of LGBTQI-phobia are thus presented*.* In conclusion, the study aimed to give visibility to the violence based on sexual orientation, gender identity or expression that takes place in HEI’s, and to identify successful practices and decisions for the eradication of this violence.

## Methods

This article has been focussed it research methodology for social impact. The voices of the end-users of the research have been incorporated in all phases of the research, in order to contribute to the social impact and social transformation of the conflict [31]. To make that possible, the methodological design is based on the Communicative Methodology of Research (CMR) [32], which stands out for its capacity to identify successful actions that contribute to overcoming inequality and to generate social policies based on these actions. CM stands out for generating scientific knowledge through the contrast of the scientific evidence (contributed by the researchers) and the contributions of the social agents’ participants in the research, defined as the *world of life* [33–35].

The study was designed to understand how, why, in which forms and circumstances this form of violence in HEI’s take place affecting university students’ life, health status and future. Given the scientific evidence on LGBTQI+ violence prevalence and the aims of the research, the main research objective is to improve the quality of life of LGTBIQ university students, breaking the silence that exists about the violence they suffer.

Following the communicative perspective, Uni4Freedom has implemented mixed-methods research [34] of which the qualitative techniques’ results are presented in this article. Semi-structured interviews^1^ with communicative orientation have composed the fieldwork of the study taking place in six universities of the Catalan region [36–38]. The fieldwork has been designed in order to, firstly, make an approach and a diagnose of the reality that the LGBTQI+ community faces at Catalan universities and, secondly, delve into the perspective of university staff and professors, exploring the possibilities to implement and propose transformative actions for the inclusion and non-discrimination. The population target of the study is the academic community enrolled in different disciplines within the project’s six partnering Catalan universities^2^: students, university professors, administrative staff and heads of equality commissions or units at these institutions.

### Communicative organization of the research

In order to ensure the social impact of the research, the voices of the LGBTQI+ community and LGBTQI+ organizations have been included in different forms and phases during the study. Their participation has been indispensable, contributing with reciprocity, advice and follow-up to guarantee that the research objectives are met and ensuring ethics’ standards in the methods. The Advisory Board is a follow-up and supervising body which has been formed by representatives of organizations of reference on LGBTQI+ rights in the territory^3^. In two different stages of the project and face-to-face meetings in 2018 and 2020, this board has debated and reviewed the methodological plan, research technics, findings and proposals grounding the materials in their experience and expertise in LGBTQI+ rights and reality. Their contribution has contributed to comply with ethical principles as well as to ensure the work and results for the improvement of the situation of the LGBTQI+ community at Catalan HEI’s.

### Fieldwork

We have conducted 12 semi-structured interviews with communicative orientation to LGBTQI+ university staff from the partner universities of the project. Besides, we have conducted 4 semi-structured interviews with communicative orientation to heads of equality units or commissions from the partner universities of the project. And finally, 12 *communicative daily life stories* with University students have also been done. These technics have followed the communicative orientation of the methodology by facilitating reflective dialogues between participants and researchers about the incidence of violence due to sexual orientation, identity or gender expression faced in their careers as university professor/staff or student. Proposals to make university a more LGBTQI+ friendly space were also gathered from these qualitative work. The distribution of research tools and participants has been as follows:

### Research tools

The guidelines of the interviews have been designed following the communicative perspective, attending to the results of the literature review and contrasted with the Advisory Committee. This combination in the design process has allowed us to develop complex guidelines that enable the identification of situations, characteristics and circumstances that either promote or allows to transform situations of violence based on sexual orientation, gender identity or expression at HEI’s. We have identified three sections that have let us build the data collection process of the interviews. In what follows, the structure of the interview under the three sections and some of the questions of the interview’s guidelines are introduced:

1. To describe and presentgeneral aspects of the research method, theme, exploring different perspectives and ideas in relation to the reality of the situation of the LGBTQI+ community in University.
2. To study the experience or perception of violence based on grounds of sexual orientation, gender identity or expression at University or spaces related to the institution according to the results of the literature review. The opening question of this section in the case communicative daily life stories with students was the following:
  - “If you know of any cases of violence due to sexual orientation, gender identity or gender expression, comment on it:
    Do you think that the people who suffer from any of these situations are considered victims of violence because of their sexual orientation, gender identity, or gender expression. Why? Why not?
    What was your reaction to the situation of violence? Why?
    What was the victim’s reaction to the violence or discrimination? Why?
    If it was reported to the university, what was the institution’s response? How did they interpret it? How does the institutional response affect the victim’s decisions and behaviors?
    What consequences did the fact of reporting have on the aggressor? And on the victim?
    Do you know what has to be done in the case of suffering a situation of violence due to sexual orientatio, gender identity or gender expression?”
3. To approach the perception of institutional strategies to detect, prevent and intervene in cases of LGBTQI-phobia by the research participants attending to their different roles in the community. Two questions from the semi-structured interviews with university staff from this third section were the followings:
  - “In the section on harassment and discrimination, the inclusion of sexual orientation and gender identity/expression in anti-discrimination policies and the approval of protocols against LGBTI-phobia is considered. Some universities, such as Oxford, Tuft and UCL, have explicit online policies regarding sexual orientation, and other ones have specific policies concerning the trans* community, as well as policies to ensure inclusive language.
    Do you think that this would be convenient at your university? Do you think it would be appropriate? Do you think it would be viable? Why? What benefits would it have and how would students experience it?”
  - “Finally, on training for members of the university community to detect, prevent and act against LFBTI-phobia. Universities like Pennsylvania and Washington train the community to ensure Safe Zones, zones free of any violence, and others like Cambridge and Oxford train the community in successful actions, such as bystander intervention and providing online resources.
    Do you think that this would be convenient at your university? Do you think it would be appropriate? Do you think it would be viable? Why? What benefits would it have and how would students experience it?”

### Ethical validation

The study has received the ethical validation of the ethical committees of Girona University and Lleida University in 2019. After submitting a detailed protocol for the fieldwork, containing consent forms and interview guidelines, the Ethics and Biosafety Committee of the University of Girona approved the start of the fieldwork. For the second ethical approval, the Committee for the approval of research studies at the Faculty of Nursing and Physiotherapy of the University of Lleida approved the fieldwork plan, consent forms and guidelines for interviews under the ethical requirements of confidentiality and good praxis without any objections.

Consent forms were systematically signed by all research participants and by the researchers implementing the tools, in order to ensure the former’s rights in the research. These forms helped us to protect their right to confidentiality, anonymity, wilfulness, possibility to stop or leave the study at any moment and receive all necessary information for their involvement by the researcher.

Aside of the institutional validation, the research counted with an Advisory Board which supervised, followed-up and advised the research team in three different phases: approval of the literature review, fieldwork plan and preliminary results of the study. This board was composed by members of the LGBTQI+ community, university students and representatives of active organizations for LGBTQI+ rights of the territory^4^. Their belonging to the targeted community was due to the need of including the voices of the end-users of the research throughout the whole process of the research. The role of the board has been to relate the theory and scientific evidence to the daily reality of the LGBTQI+ community, reinforcing the transformative role of the research through their very contributions in the study.

### Data analysis

The analysis chart has been designed to collect contributions from interviews and communicative daily life stories considering all the dimensions and categories selected (Table 1 – Result analysis chart). Dimensions are located in the rows and refer to the two sorts of results depending on their contribution or transformation of the target conflict, referring to the Communicative Methodology. Categories are the concepts that are being used in the research process to analyse the results of the fieldwork and they are located in the columns (Table 1 – Result analysis chart). The categories have been defined through a deductive method of definition, meaning that they have been determined before fieldwork through the study of scientific literature regarding LGBTQI-phobia in HEI’s. The categories resulting from this study are: LGBTQI-phobic violence, actions against the violence and university politics and measures against the violence.

**Table 1:** Result analysis chart

	Violence against the LGBTQI+ groups	Actions against the violence	University politics and measures
Exclusionary dimension	1	2	3
Transformative dimension	*4*	5	6

Chart of relation between categories and dimensions for the analysis of the results

From: Table created by the research team of the project during the elaboration of the results for the analysis of the results of the fieldwork

The research team has processed the qualitative results of the fieldwork ensuring the anonymity of the participants in all the phases of the study. Members of the research team have transcribed the interviews and daily life stories verbatim. Then, the research team has coded the transcription by using the numbers of the designed analysis chart (Table 1 – Result analysis chart), identifying results and matches between the targeted categories and dimensions on the transcriptions. We have not made use of any software or program for the systematization of this process.

## Results

In this section, we have made an in depth approach to the research target: studying violence against LGBTQI+ community in HEI’s. On the one hand, we have analysed the results that do not contribute to overcoming the problem of violence based in sexual orientation, gender identity or expression, encompassed within the exclusionary dimension. On the other hand, we have analysed the contributions that have an influence in transforming and overcoming the targeted violence in HEI’s, included in the transformative dimension. All of the results presented belong to the research tools and the research participants already mentioned in the fieldwork subsection (Table 2 - Fieldwork distribution).

**Table 2:** Fieldwork distribution

Research tool	Participants	Institutional affiliation
Semi-structured interviews	12 active university professors or staff.	Belonging to the participant Universities in the study^5^
Semi-structured interviews	4 heads of the University Equality Unit or Committee presently active, and professionals working in them.	Units belonging to the participant Universities in the study.
Communicative daily life stories	12 university students starting from second year of the BA degree up to the last year of the PhD.	Belonging to any Catalan University.

Chart of the distribution of research tools, sample and target institutions for the organization of the fieldwork

Form: Table elaborated by the research team of the project for the organization and distribution of research tools and academic community

### Violence’s normalization and internalization

Normalization and internalization of LGBTQI-phobia are the most present consequences of the violence in HEI’s. These results show the need to promote measures of awareness-raising to promote respect to diversity. Under this category, we highlight the normalization of violence in the daily discourses at universities as a consequence of the constant violence against the LGBTQI+ community. The normalization of violence is funded on naturalizing discriminatory comments towards the LGBTQI+ community, which can happen even within classrooms, as stated by a LGBTQI+ university professor in an interview:

Then, inside the class, let me think... at the break and when we leave and so on, I’ve seen someone say to another "hey faggot, you didn't get the work done today!" maybe they said that and, I don't know, I have it so incorporated that I don’t realize either.

In this sense, students have also shared in everyday life stories experiences that prove the naturalization of discriminatory discourses towards the LGBTQI+ community, as stated by a female undergraduate and LGBTQI+ student in a communicative daily life story:

Well, I don’t know, if in class or between classes, we are talking, or they are talking, so in a group, and they want to refer to a boy as being a freak or weaker than the rest they refer to him as a faggot.

The LGBTQI+ participant students in the research have claimed the consequences of the normalization of the violence. Following their discourse, they have found that reproduction of homo and lesbo-phobic comments and the self-internalization of the violence are results of having received a LGBTQI-phobic socialization. As a female and LGBTQI+ student expresses in a communicative daily life story:

Many times, I think they overlook these comments because we are used to them. For me what happens to me is like, if one day I hear someone say butch or something, it's not hard for me to pass but I guess I would think that he’s an asshole, you know? But then I would think that, he’s silly and that's it and I wouldn't take it as something personal, but as something more social that looks normal.

### Transgender vulnerability in the conflict

Research has shown that transgender people are the most prone to have difficulties and to suffer violence or discrimination at HEI’s [4–6]. This form of vulnerability in the university context is even more disturbing when the results show the complexity and accumulation of forms of violence that only transgender students suffer. There can be specific circumstances that transgender students live, such as the social transition and the bodily changes, elements that can make their educational process at university even harder when belonging to the LGBTQI+ community. As a transgender student states in a communicative daily life story regarding the transitioning:

Then I made the transition and it's like that, with the medication and that, I was like super confused with many things, I was relocating mental issues, because in the end I didn't know many things either, because the medication numbed me and I don't know. Of course, I did notice suspicion and misunderstanding and a feeling of being something weird, feelings of disgust, by some colleagues and I realized it but well, as I’m saying I tried to ignore it because I have enough problems.

The exclusionary discourses, looks and refusal perception is clear in the voice of the interviewed people, showing the need of promoting measures of awareness raising that advocate the respect to diversity and differences. In this sense, the need of intervention and respect towards the transgender groups is especially relevant, as it has been shown in the interviews’ fragments.

### Unfamiliarity of institutional mechanisms and interventions

Secondly, findings on *university policies and measures* have indicated the lack of actions, university policies and measures to fight violence and, at the same time, they prove the ignorance of professors and staff about the mechanisms to prevent and intervene in cases of LGBTQI-phobia. Furthermore, the lack of cases of violence due to sexual orientation, gender identity or expression reported at equality offices indicates the complexity of this form of violence and the likely unawareness about violence based on the grounds of sexual orientation, gender identity and gender expression by officials at universities. The fact that some Heads of University Equality Offices claim not to have received complaints regarding violence based on sexual orientation, gender identity or expression is relevant, as one experienced worker on an Equality Office shared regarding the cases of LGBTQI-phobia in an interview:

The truth is that no. I have not dealt with any cases at the observatory, no petitions nor expositions have been received of violence based on gender identity or sexual orientation. We haven’t realized it. For me, it hasn’t come directly to me as a teacher or as a colleague. It hasn’t reached me. I know it’s a college reality, but the truth is I can’t say it’s a reality for me because I haven’t seen it.

The figure ‘0’ of cases of violence based on sexual orientation, gender identity or expression at universities can be explained by the lack of mechanisms and abilities by university professors and staff to identify and detect the violence [6, 39, 40]. Moreover, it could also be justified by the attempts to generate safe and friendly spaces for the LGBTQI+ community to make the process of filing a complaint of LGBTQI-phobia easier. As we can see in the following fragment from a communicative daily life story with a transgender student which has already faced the process of name change, he reflects on other possibilities to it within HEI’s:

I think that a trans person should not go through an equality unit to request a name change, right? But I think that this could already be done in a much easier administrative process of administrative, that is, how you do your... You fill out your application for the first time, that is, in that database, what if there were what is called a chosen name?

The lack of knowledge from university professors, staff and officials about measures, resources and officials of reference in cases of LGBTQI-phobia has been stated in the interviews as a constant reality, as mentioned by a female university professor in an interview: “I’m not responsible. I don’t know if within the management team there is someone in charge of this policies in case there is a problem.”

We have identified other indicators apart from unawareness which could respond to the lack of a support network for victims of LGBTQI-phobic violence within HEI’s. Many university policies and educational protocols for the prevention and intervention in cases of violence based on sexual orientation, gender identity or expression have been developed in the last years from Equality Units and Commissions and other spaces towards equality and against discrimination in HEI’s. The ignorance of the international scientific evidence about the existig policies carries a limitation in the struggle against the violence towards the LGBTQI+ community. This is due to the lack of knowledge and training on the measures and the roots of LGBTQI-phobia for the implementation. In order to understand the notions on institutional measures to intervene in conflicts based on sexual orientation, gender identity or expression, we can see an active LGBTQI+ university professor’s discourse where he discusses the thoughts on the transgender name-change process as it follows a fragment of an interview:

The doubt that I was holding is the legal part. Without a doubt the university has to support straight away and if it is necessary to change, it is changed [referring to the name], if you have special needs, it has to be attended, they have to be listened to and we have to see what can be done, of course. What confuses me a little is the legal issue. (…) To the official lists, they appear with the birth name, but they can be changed, and it seems viable, and they are comparable because at the end, that’s the name that they identify with. “I do not identify myself with Antonio José... I identify myself with Toni.” And it seems very comparable. If this person wants to change the name of Maria to Peter because he identifies himself as Peter, so Peter be it and that’s it. What I find most complicated is at a more internal level, for example in the records, that you have the name changed because there would probably be a conflict of legal identity.

In this sense, students agree in recognizing that ignorance complicates the process of identification and support in particular situations of LGBTQI-phobia at universities. For that reason, training and awareness raising on LGBTQI+ issues are both considered very necessary towards turning all university members into agents of change, whether being or not part of the LGBTQI+ community, as a cis-heterosexual female student points out in a communicative daily life story:

I think so, I have not experienced these situations, and I don’t know these type of situations. I'm sure it happened. I think that it should be known both for those who do not know it and for those who suffer it or have seen it, to know that they are not alone that someone is aware of the issue and that they take measures against these situations and that there are those points of help. There are also people who do not want to come out of the closet and they may have problems but they will not ask for help because they have not yet come out of the closet, so it would be good for them to know that there are actions that can help them without anyone knowing anything and keeping their secret. It is an option for those people to have help.

### University as a safe space

Secondly, on the variable *LGBTQI+ actions against violence* findings point at the existence of three protective factors that lead to overcoming violence and discrimination: HEI’s perceived as safer spaces compared to other places, compromise and predisposition of professors to successfully prevent and intervene in cases of violence and university protocols and measures of intervention including all university community. This is due to the role of Equality Units, their familiarity, respect and openness has an important effect in the prevention and intervention of cases of LGBTQI-phobia. We have identified that HEI’s offer a very wide window of possibilities for intervention, acceptance and respect compared to other spaces, as a LGBTQI+ female student points out in a communicative daily life story:

Sexual diversity is more comfortable at university than in other places and that’s why I also think it’s sometimes easier to make more demands within university, right? Because as there is this freedom or this friendly climate, right? Friendly to make claims, to make demands for improvement, so it’s easy to get it and therefore I think that precisely freedom encourages more freedom of expression, right? And more diversity.

Another protective factor within HEI’s towards the LGBTQI+ community and for the transformation of the violence and discrimination is related to the compromise of professors to prevent and intervene. The alliance between students and professors is especially valuable when having the support of a more powerful group within the educational institution in terms of decision and action. This particular support can be offered for different reasons, firstly for the training, awareness and activism in terms of rights by professors. Next, the importance and urgency of intervening in order to transform and stop the conflict based on gender or sexual diversity is made explicit by one of the LGBTQI+ university professors interviewed:

Having just one victim is enough to talk about it and explain that these things are happening, anonymously. If not, we have to orient ourselves differently, lead it in a way that if things happen socially, we try not to let them happen here. Obviously, they shouldn’t take place anywhere. We protect the space; I think we have to find a balance in that so as not to create an alarm.

The compromise to intervene in cases of violence based on sexual orientation, gender identity or expression has been expressed in different forms in the discourses of LGBTQI+ staff. The following case goes one step further as, aside of an open commitment with LGBTQI+ issues, this social conflict is taken into consideration as part of the very teaching praxis. As a result of this, we see how a safer space in the classrooms can be created, by making sexual and gender diversity an issue in the lectures. A LGBTQI+ male professor - committed to openly talking and discussing about matters of gender, sexuality and diversity in class-, talks about the reaction of his students when addressing these issues:

No! Not in class, maybe that's because we criticize it, and make people think and everything is politically correct...to let them see their experiences based on that and then see how they act...of course, in class I guess that they are aware that it would not look very good for them to do joke about it if we are working for them not to do so in their own environments.

When breaking the silence on the issue of LGBTQI-phobia so that the topic becomes a recurring theme in the classroom, students become active upstanders questioning themselves and intervening in cases of violence [41]. In this sense, another cis-heterosexual university professor highlighted in the interview the need to break the silence and generate mechanisms facilitating that people dare to complain:

It may also be that things are happening and we don’t know it because there aren’t protocols, so this is also a way to encourage people who are going through things to report it. Because violence is always hidden actions. If this is giving them a little encouragement to report and explain what is happening, even if the violence is not physical, that is verbal, that is behavioural, exclusionary...

### The value of receptiveness and alliances

Thirdly, on the variable *university policies and measures,* we have identified evidence of the openness towards sexual and gender diversity by university professors, also considering the need of prevention and intervention plans and measures of high quality in order to transform the reality. This is the case of protocols and measures of intervention generated by the Equality Units at HEI’s that have been interviewed. They highlight the quality and connection with the reality of the LGBTQI+ community of their regulations and intervention measures in their own Units. This is due to the success of negotiation processes between HEI’s and Equality Units, thanks to the sanctions that exist in case of not implementation and to the inclusion of gender identity and sexual orientation perspectives in the regulations. This is introduced by a long experienced worker on the service of the Head of an Equality Office at one participant university in an interview:

We have a regulation for the prevention of gender violence. The difference between regulation and protocol is that all other universities have protocols, ours has sanctions. The others do not have it typed. Our regulation -which was one of the first to be done, but which had two years of negotiation with the University-, is a comprehensive one because it covers the entire university community (officials, staff, professors, students) and it is also a one that entails penalties that many of the university’s protocols do not have. Then what we have done is the adaptation of it, when we already made the regulation, we put for example everything that was harassment due to sex and sexual orientation, we added all the sexual orientation tag.

The interviewees have shown willingness to learn the measures and implement them in the Catalan University contexts, even though if they have not received any training in LGBTQI+ issues. The following fragment refers to a LGBTQI+ male university professor’s interview referring to the measures of trans-inclusion at their institution:

Of course, as the number does not change, so there is no problem, and everything is linked to the ID number and instead you can change the name. I think it’s ok, if there is a real need for it and it is a request from individuals or the community itself, I do not see it difficult and do not see a problem. I think it would bother me to call this person by the name with which they do not feel identified. If they tell me to change the name, I tell them that way, because otherwise there is not an effective dialogue, so I think that if possible, I think it’s perfect and go ahead.

Predisposition and interest by professors have appeared in the qualitative fieldwork together with the claim of needing scientific evidence as well as the inclusion of voices of the own community and of experts in the field. Then they could advise and orientate regarding measures and politics at HEI’s. A university professor claimed it during the interview as follows:

Totally, but I think that the experts here are somehow the ones who have to take the lead because I do not consider myself an expert on the subject, I am a total ignorant, because I find it hard to find the right words to talk about this community, if we are referring to differentiated groups. Mm, I feel I can talk about certain things, but when I think about it, I think that maybe I had not realized it. My normal life is not affected, but there might be other people’s life who is (…) and then if the need exists and the university has the measures to make it feel normal, so that this becomes of normality, then I will be happy to follow any training that is needed because for me it is also an exciting topic, not morbid-like, but to know. Because it is becoming more and more visible.In the same way that this professor commented, another student also claimed the need of measures and politics to have some support in case of suffering violence based on sexual orientation, gender identity or expression. The following statement is a fragment of LGBTQI+ activities and university student’s communicative daily life story, reflecting clearly on the need of feeling institutional protection in order to feel integrated at University:

That people feel safer, better, that they have a real moral and psychological support because until now, they are not considering themselves part of anywhere. Having such a policy would help us a lot to feel that we belong and that we are considered part of something because, of course, we are having to face these LGTB-phobic behaviours and they have to be counteracted with something, right?

## Discussion

Our study demonstrates the urgency of the conflict taking place at HEI’s on the grounds of sexual orientation, gender identity and gender expression. It has also provided us with the scientific evidence and the protagonists’ discourses by bringing to discussion how both dimensions match and complement each other when facing conflicts, needs and discrimination based on sexual orientation, gender identity and gender expression. Furthermore, the study has added relevant knolwedge to previous research with an evidence-based approach and successful cases to improve protocols and strategies for the struggle against the problem of LGBTQI-phobia taking into account the voices of the university community in the Catalan context. Contributing to the previous research on gender violences at Catalan and Spanish universities [31], our study has gone further in studying the problem of gender violence against the LGBTQI community in HEI’s for the first time in Catalonia. The complementarity of both the generation of evidence and new proposals of avenues for the improvement of current protocols, policies and measures towards the inclusion of the LGBTQI+ community sets a precedent on how to turn HEI’s into more LGBTQI+ inclusive institutions.

### Challenges in identifying violence

There is plenty of evidence about how the conflict of violence based on sexual orientation, gender identity or expression is a current reality, which is visible and has become a relevant subject for the development of international policies and agendas in Europe [42]. Furthermore, the scientific literature describes how this conflict can permeate social institutions, affecting subjects in different spaces, dimensions and degrees. The main challenge identified in the struggle against this social conflict is its identification and detection in institutions, as it has been mutating and changing its form to avoid being detected [6].

Our study has, not only proven the existance of a variety of forms of direct violence that take place in HEI’s, but it has also identified more subtle and unnoticed forms of violence. Verbal forms of violence, such as homophobic and transphobic comments and jokes, paintings and non-inclusive spaces and classrooms are just the more apparent forms of violence pointed out in the research [9, 10, 13–16, 18]. What has made the situation in the Catalan context more complex is the generalized reaction of internalization shown by LGBTQI+ victims and other agents. As the literature highlights, this response of normalization and naturalization of the violence against sexual or gender diversity contributes in the reproduction of the violence against the LGBTQI+ community [3, 16, 23–25].

Besides the reaction of the victims and the LGBTQI+ community, the responses from the rest of institutional agents facing the conflict are especially significant. As mapped by the research, the role of other students and peers [27], professors and staff is key when approaching the cases of LGBTQI-phobia at university, as the relationality, authority and influence is compelling [8, 20–22]. As the findings have shown, their availability, openness and attitude towards the LGBTQI+ community and sex and gender diversity can have an impact on the perception of classrooms and university campuses as free and safe spaces. Additionally, we have found how previous debates or workshops of LGBTQI+ issues at university classrooms can prevent some forms of LGBTQI-phobia from happening. As well, these previous experiences on discussing gender could facilitate processes of social transition, reception of reports of violence or discrimination, improving the perception of the university as a friendlier and safer space. In addition, the heads of Equality Units’ figure entails two different roles: as social agent and worker as well as a representative in terms of equality and non-discrimination in the institution. In any case, this readiness and preparation does not suffice while numbers show that there are no cases of LGBTQI-phobia arriving to institutional instances in some of the participant HEI’s.

### Damage on wellbeing of LGBTQI+ students

Given the findings about the prevalence of violence on the grounds of sexual orientation, gender identity and gender expression in Catalan universities, the negative impact on the well-being and life quality of LGBTQI+ students is a fact. If the existence of violence against sexual and gender diversity in university spaces is a reality, the probability of having students suffering physically and mentally, presenting symptoms of depression, anxiety and various forms of physical ache are a worrying reality for the institution [9, 13, 16]. Furthermore, this difficulty affecting only a group of students would generate a gap in the access, quality and academic success compared to the rest of the community due to their health status [25]. The lack of social cohesion within the university community and students, has a high impact in the present and future of LGBTQI+ students in Catalonia. LGBTQI+ stigmatization also results in impediments for LGBTQI+ students to reach the same academic level and success than the rest of the students [19, 23, 43, 44].

The findings about the damage of the LGBTQI+ students at University has given visibility to the risk that the LGBTQI+ community suffers which also affects their wellbeing. This evidence indicates that the generation of successful strategies to prevent, detect and intervene in cases of violence on the grounds of sexual orientation, gender identity and gender expression is urgent. This emergency lies on the institutional duty of offering quality higher education for everyone without of any type of discrimination. In order to eliminate any form of discrimination effective anti-discrimination strategies based on scientific evidence need to be developed.

### University policies and measures a the LGBTQI-phobia

Given the results about the implemented strategies to fight and prevent forms of violence on the grounds of sexual orientation, gender identity and gender expression, issues on the evaluation, quality and follow-up of these measures are still pending. The evidence shows that the current strategies to fight, prevent and intervene in cases of LGBTQI+ realities in Catalonia are configured as responses to concrete and specific situations. This conception of the LGBTQI+ reality as a transitory conflict and circumstance implies that the forms of intervention planned only take into account the specific conflict, without paying attention to a reality that is present in all the spheres of the university. This can respond to the lack of continuous and more transversal actions that educational systems implement to carry out more equitable actions for the inclusion of LGBTQI+ realities within HEI’s.

Giving visibility and raising awareness of the LGBTQI+ circumstance is a very pressing issue present both in the literature and in the results of the research, as it can prevent different forms of LGBT-phobia. These actions are identified as protective factors in the prevention and intervention of discrimination and violence on the grounds of sexual orientation, gender identity and gender expression, as well as for generating and imporving the LGBTQI+ students’ feelings of belongingness to HEI’s. More in depth, literature and participants have identified the need of training professors and other university staff about LGBTQI+ perspective [1, 28]. This could have an impact on the way conflicts and discrimination on the grounds of sexual orientation, gender identity and gender expression is managed with professionals, ensuring safe follow-up and accompaniment processes by educators and staff -who would be trained on the situation of the LGBTQI+ community through scientific evidence-.

In order to translate this process of making LGBTQI+ issues a closer reality to the university community, the inclusion of literature from a LGBT+ perspective in the academic curriculum has been identified as having the highest impact for the reduction of violence and discrimination on the grounds of sexual orientation, gender identity and gender expression [20, 28]. Its implementation would require HEI’s to include LGBTQI+ issues horizontally in all university degrees’ classrooms, considering as an institutional duty the need of ensure freedom of living and expressing sexual and gender diversity. Another way of institutionally protecting the LGBTQI+ community is by explicitly supporting the community as has been informed by the literature [6, 9, 29, 30]. Lastly, the urgency of addressing the transgender issues at universities is present in both the project’s results and literature [45], highlighting the need of articulating successful practices and accompanying processes to transgender students for the improvement of their health [43]. This would require that universities start conceiving the transgender reality as a continuous, individual and changing phenomenon that goes beyond the name and gender change in the identification documents, affecting the live of students with different intensity and in different stages and social circles.

Altogether, the complexity of assessing and reviewing the success and impact of university protocols, measures and strategies to intervene is both a scientific and socio-political issue, attending to the changing political circumstances that affects the European and Spanish context. The research limitations that have affected the study have been closely related to the human interactions during the fieldwork, the protection of the anonymity, the search for gender and sexual minority participants and the issues of visibility and public recognition of participants. For that reason, we have worked very hard in the ethical framework and approval to ensure everyone’s safety, respect, confidentiality and support during and after the fieldwork. Otherwise, the qualitative method and results of the study have also set limits on the applicability and transferability of the findings. Although quality-centred findings do not offer results that can be generalized to the whole Catalan university community, they have allowed us to delve into the reality of the Catalan HEI’s through the discourses of university staff and students.

## Conclusion

Following our research goal of improving the quality of life of LGBTQI+ university students, through the CM and breaking the silence that exist about this sort of violence, the study identified protective and exclusionary factors likely to have a high impact in the quality of life of University LGBTQI+ students in the Catalan region. This innovative and transformative focus has provided the dialogue-based methodology on the study of the conflict of gender and sexual diversity in the most relevant educational institution.

The need of studying the conflict of LGBTQI-phobia at universities lies on the importance of higher education in the lives of students and in their future possibilities. It also falls on the strong impact of suffering violence and discrimination for several years while the right of living one’s sexual orientation, gender identity and gender expression freely is not guaranteed. All of this shows how gender and sex norms permeate educational institutions [46, 47], making visible the current positioning of Catalan HEI’s against LGBTQI-phobia and towards a more inclusive and diverse university community.

New avenues and research targets on this matter could contribute to identifying other needs and axes of actions that could be essential in the struggle against LGBTQI-phobia. On the one hand, investigating the positioning of professors in the classrooms and their previous training on gender and LGBTQI+ perspective with a base on scientific evidence could open new lines of research for the prevention of violence. Furthermore, the inclusion of LGBTQI+ literature to be addressed in classrooms and the impact of normalizing gender and sex diversity in educational institutions would also be relevant. Our study highlights the importance of the alliance of university professors in the struggle against LGBTQI-phobia as upstanders in the conflict, an issue that must be paramount in new research lines and actions against violence on the grounds of sexual orientation, gender identity and gender expression. On the other hand, studying the case of transgender needs and trajectories in HEI’s is still a pending issue. Tackling the need to underst transgender identities and non-binary gender expressions within the institutional framework would contribute to detect and explain forms of violence yet to be identified as well as the strategies to counter these.

## Data Availability

The datasets used and/or analysed during the current study are available from the corresponding author on reasonable request.

## Abbreviations

CM: Communicative Methodology
HEI: Higher Educational Institution
LGBTQI+: Community of lesbian, gay, bisexual, transgender, queer, intersexual and other groups with non-conforming and dissident identities, orientations or expressions

## Footnotes

1 Interviews had an approximate duration of 40 min and the place was chosen by both participant and researcher. Interviews were normally implemented in public spaces which allowed the researcher to generate an atmosphere of trust, such as cafeterias or university offices. The materials result of the interviews were the manual transcriptions made by members of the research team of the Uni4Freedom project. Then, the analysis took place as it follows in the Data Analysis subsection.

2 The partner universities of Uni4Freedom are: Rovira i Virgili University; University of Barcelona; University of Girona; University of Lleida; Ramón Llull University and University of Vic.

3 Participant entities and organizations on the Advisory Board:

- Col·lectiu H2O · Gais, Lesbianes, Bisexuals i Transsexuals del Camp de Tarragona.
- SAI Tarragona - Servei d’Atenció Integral a les Diversitats Sexuals i de Gèneres de Tarragona.
- Xarxa Solidària de Vctimes de Violència de Gènere a la Universitat.
- Gènere Lliure.
- Col·lectiu de gèneres i sexualitats dissidents (GSD).

4 The participant entities in the Advisory Committee have been: - Col·lectiu H2O (Gais, Lesbianes, Bisexuals i Transsexuals del Camp de Tarragona). - SAI Tarragona - Servei d’Atenció Integral a les Diversitats Sexuals i de Gèneres de Tarragona: suport social, informació i atenció piscològica i jurídica.

5 The participant universities in the study are:

- Rovira i Virgili University.
- Girona University.
- Lleida University.
- Vic University.
- Barcelona University.
- Ramón Llull University.
